# Inpatient’s, therapist’s and staff’s expectations regarding treatment and their effects on placebo response in the psychiatric ward – results from an add-on oxytocin RCT

**DOI:** 10.1007/s00213-024-06593-x

**Published:** 2024-07-25

**Authors:** Uri Nitzan, A. Grossman-Girron, O. Sedoff, H. Maoz, O. Arad, E. Tilbor, C. Dror, D. Tzur Bitan

**Affiliations:** 1 Shalvata Mental Health Center, 13 Aliyat-Hanoar st, Hod-Hasharon, 4534708 Israel; 2 School of Medicine, Tel-Aviv University, Tel-Aviv, Israel; 3 Department of Behavioral Sciences, Ariel University, Ariel, Israel; 4 Department of Community Mental Health, University of Haifa, Haifa, Israel

**Keywords:** Expectations, Anxiety, Depression, Inpatient, Oxytocin, Placebo

## Abstract

**Objectives:**

Patient’s and therapist’s expectations are considered an important factor influencing placebo response in experimental and therapeutic settings. Nevertheless, the placebo effects of common neurological facilitators that promote treatment efficacy have not been explored. In the present study we examined the estimations of patients, therapists, and staff members, regarding their treatment type and assessed their influence on the facilitating effects of oxytocin.

**Methods:**

Patients (*N* = 87) were randomized and double-blindly allocated to receive either oxytocin or placebo, twice daily for a period of four weeks, as part of a larger randomized, double-blind, placebo-controlled trial. Patient’s, therapist’s and staff’s expectations were assessed based on their estimation of treatment type (agent or placebo). Multilevel modeling and univariate and multivariate regression analysis were performed to assess the effects of patient’s, therapist’s, and staff’s estimations on treatment outcome beyond the effects of treatment type.

**Results:**

Staff’s, therapist’s, and patient’s estimations were significantly associated with treatment outcomes. Nevertheless, only therapist’s and patient’s estimations significantly predicted improvement beyond actual administration, with therapist’s and patient’s estimations associated with improvement in trait anxiety *(STAI-T, B=-1.80*, *p* < .05, and B=-2.02, *p* < .05, respectively); therapist’s estimations were associated with improvement in general distress *(OQ-45, B=-3.71*, *p* < .05), and patient’s estimations were associated with symptom relief *(HSCL-11, B=-0.13*, *p* < .05). Overall, patient’s estimations had a higher relative contribution to treatment success, with standardized coefficients across scales ranging from − 0.06 to -0.26.

**Conclusions:**

The neurobiological factors that promote treatment success are also influenced by patient’s and therapist’s expectations. Future studies should consider these effects when examining their impact in inpatient settings.

## Introduction

Patient’s and therapist’s expectations are known to affect the outcome of clinical trials(Rutherford and Roose 2013; Doering et al. 2014; Bingel 2020). Previous studies demonstrate that expectancy has a profound influence on the placebo response and that its neuroanatomical and neurophysiological sequalae are evident across various disorders (de la Fuente-Fernández et al. 2001; Petrovic et al. 2002). In fact, expectancy is considered one of the central theoretical approaches to understand the mechanism of the placebo response, especially in the treatment of pain and patient healthcare of psychiatric disorders such as depression and anxiety (Sotsky et al. 1991; Sonawalla and Rosenbaum 2002; Rutherford et al. 2010b).

Clearly, patients who know, or at least believe, that they are receiving active treatment have a higher level of expectancy, reinforced by the belief that a scientifically validated intervention will lead to positive outcomes (Benedetti et al. 2003; Rutherford et al. 2010a). Awareness of receiving active treatment can further enhance the effectiveness of the treatment itself, potentially leading to increased symptom reduction and improved overall well-being (Kirsch 2003; Kaptchuk et al. 2008).

Numerous and diverse studies point out the role of expectancy in determining the extent of the placebo response in clinical trials. Patient’s expectations levels was found to significantly predict response to antidepressants medication (Meyer et al. 2002; Krell et al. 2004). Furthermore, the number of treatment arms in antidepressant clinical trials was found to correlate positively with the magnitude of the placebo response, with higher likelihood of receiving active medication generating higher placebo response (Khan et al. 2008). Additional studies indicate that antidepressant response is weaker in placebo-controlled trials than in comparative open-label trials in which patients know they are receiving one of two medications (Kim and Holloway 2003; Roose and Schatzberg 2005; Sneed et al. 2008; Rutherford et al. 2009).

Therapist’s expectations have also been found to play a significant role in shaping patient’s placebo response (Gracely et al. 1985; Kaptchuk et al. 2008). The therapist’s belief in the efficacy of the treatment, as well as his expectations, have shown to influence the placebo response and to facilitate symptom reduction (Glick and Margolis 1962; Jacob 1971; Forester 1999). It has also been demonstrated that rater expectations, and rater-patient relationships can impact the placebo response in clinical(Williams et al. 2012; Leuchter et al. 2014). Similarly, the therapeutic alliance between research personnel and the and between therapists and patients are an important factor in determining expectancy levels regarding treatment and placebo response(Kirsch 2004; Khan et al. 2012; Williams et al. 2012; Leuchter et al. 2014).

Although patient’s and therapist’s expectations have been found to influence therapeutic outcomes, not much is known of these effects in inpatient settings, where the primary caregiver is part of a large and diverse staff likely to possess different perceptions and expectations (Bodner et al. 2011, 2015a). The present study, conducted as part of a randomized, double-blind, placebo-controlled trial, aimed to examine whether patient’s, therapist’s, and staff’s estimations regarding their treatment type (i.e., Oxytocin (OT) vs. placebo) were associated with therapeutic gains over and above the actual therapeutic effect (i.e., allocation to OT vs. placebo). Furthermore, we aimed to assess the relative contribution of each placebo effect (patient’s, therapist’s, and staff’s) over and above the active treatment. Based on previous literature, we hypothesized that patient’s, therapist’s, and staff’s estimations will significantly influence treatment outcomes beyond actual assignment to OT or placebo. We also hypothesized that patient’s estimations will have the strongest impact on treatment outcomes.

## Method

### Participants

For a full description of the participants and RCT design, see Grossman-Giron et al(Grossman-Giron et al. 2023). To briefly summarize the relevant information, eighty-seven inpatients completed the study between July 2018 and November 2021 in the inpatient wards of the Shalvata Mental Health Center, Hod-Hasharon, Israel; forty-three inpatients were randomized and double-blindly allocated to receive placebo and forty-four were double-blindly allocated to receive OT. Furthermore, the routine interaction between patients and the staff included 2–3 sessions of psychotherapy and a weekly meeting with a psychiatrist. Additionally, there were routine interactions between the patient and the nursing staff (distribution of medication, reassurance, etc’) and the occupational therapists (Functional assessment, rehabilitation, etc’), and group therapies with various staff members.

The mean age of the patients was 34.44 (SD = 16.26) and 71.3% were female. Regarding diagnosis, 17.2% were diagnosed with anxiety disorders, 55.2% with affective disorders, and 27.6% with a personality disorder. No significant differences were found between the active and placebo groups in terms of age, sex, and diagnoses. Inclusion criteria included adults 18 years of age or older; diagnosis of a psychiatric disorder of any type made by the treating psychiatrist using DSM-5 diagnostic criteria; expected duration of hospitalization of at least four weeks. Exclusion criteria were pregnancy as indicated by positive bHCG levels; evidence of required electroconvulsive therapy; comorbidity with substance abuse; Autistic Spectrum Disorder (ASD), psychosis, or mental retardation spectrum disorders; potential suicide risk.

### Treatment and assessments

The protocol of the study was approved by the IRB of the Shalvata Mental Health Center. It was also preregistered in the Israeli Ministry of Health database (reference number: MOH_2017-12-05_002003) and at clinicaltrials.gov (identifier: NCT03566069). Consolidated Standards of Reporting Trials (CONSORT) guidelines were followed during the study. All patients signed informed consent before participating in the study. Patients were randomized and double-blindly allocated to receive either oxytocin or placebo twice daily for a period of four weeks as part of a larger randomized, double-blind, placebo-controlled trial. Two independent nurses were responsible for computer-generated randomization with a 1:1 allocation ratio. The two nurses had no association with the study or ward staff. Both oxytocin and placebo were delivered while identified by patient name only, to insure treatment masking. The two nurses had no association with the study or ward staff. OT\placebo was inhaled in two sprays, one into each nostril (8 IU into each nostril), according to standard guidelines [16].

Patient’s baseline scores on all measures were obtained. The primary outcome measures included: The Outcome Questionnaire-45 (OQ-45), a self-report measure aimed at evaluating patient’s outcomes during therapy, used to assess symptom distress, interpersonal relationships, and social role performance. This scale includes 45 items, with higher scores indicating higher subjective distress related to symptoms, more problems in interpersonal relationships, and higher dissatisfaction and inadequacy in social role performance; The Hopkins Symptom Checklist–Short Form (HSCL-11), a brief self-report measure of symptomatic distress, including 11 items, with higher scores indicating higher symptom severity. Secondary outcome measures included: The State-Trait Anxiety Inventory (STAI), used to assess state anxiety and stable aspects of anxiety tendency, with two separable subscales comprising of 20 statements for each dimension. Responses were rated on a 4-point Likert scale, with a sum ranging from 20 to 80, with higher scores indicating greater anxiety for both state and trait anxiety; The Hamilton Rating Scale for Depression (HRSD), a semi-structured clinical interview, used to assess the level of depression, containing 21 ratings measured on three (0 to 2) or five (0 to 4) point scales. The sum of the HRSD items ranges from 0 to 52 points, with higher scores indicating higher depression severity.

A side-effects profile was evaluated weekly via a self-report measure, which was developed for the purposes of this study. The scale was aimed at assessing potential side-effects of IN-OT, in accordance with those found in previous studies (MacDonald et al. 2011). It included 18 items as a list of possible side-effects, such as “having a headache, dizziness, shortness of breath, muscle pain, etc. The patient was asked to rate each item on a 3 points scale ranging from 0 (I did not feel this) to 2 (I felt this more than usual)”.

During the 4-week study, patients were assessed for anxiety and depression 2–3 times per week and for stress and side effects once per week. All measurements were taken again at the end of the study intervention. At the end of the study, following the last treatment, the therapists, and the medical staff (i.e., the attending psychiatrist and nursing staff members) were asked to share their estimation regarding the group assignment of each patient, i.e., whether the patient received the active substance (oxytocin) or a placebo in their appraisal. The following question was presented to the therapists and staff: “Please state your guess regarding the treatment conditions: Do you think the patient received OT or placebo throughout the intervention?” Patients were asked the same question, “Do you think you received OT or placebo?” If they answered that they did not know, they were advised that the answer should be based on their best guess. Based on previous literature, this served as a reliable measure of expectations about treatment efficacy and study outcomes (Sotsky et al. 1991; Brown et al. 2000; Khan et al. 2008).

Perceived group assignment included responses from 41 therapists and 47 psychiatric staff members (38 nursing staff and 9 psychiatrists) overall. The therapists were psychiatrists (*n* = 13, 32%), psychologists (*n* = 6, 15%), social workers (*n* = 5, 12%), and interns (*n* = 17, 41%). The medical staff members included those present at the time for each intervention period, as the nursing staff administered the drug and closely monitored the inpatients as part of their ongoing workload. For each patient, the perceived group assignment response rate from overall medical staff members ranged between 2 and 14 responses per patient, due to staff member’s inconsistent availability. This resulted in a total of 608 perceived group assignment from medical staff members, across all patients.

### Statistical analysis

In order to examine the effects of patient’s, therapist’s and staff’s expectations on therapeutic gains, multilevel modeling (MLM) analysis was employed. These models were aimed to create the outcome variable of improvement across all measures. As previous analyses of the outcomes in this study showed no significant therapist’s effects, as indicated by relatively low intra-class correlation across all measures (ICC, largest ICC was 0.004), models were estimated as two-level models, with restricted maximum likelihood estimation (Grossman-Giron et al. 2023). To obtain the slopes of improvements across all outcome measures, time was entered as Level 1 fixed and random effect. Slopes were then saved and subsequently served as outcome variables in a series of univariate and multivariate linear regression analyses. The analyses followed three stages. First, we examined the overall effect of patient’s, therapist’s and staff’s expectations using univariate linear regressions. Secondly, we assessed the relative contribution of each type of placebo effect (patient’s, therapist’s and staff’s) on therapy outcomes using multivariate regression analyses. Finally, we explored whether the effects sustain after controlling for actual administration using multivariate regression anaylses. Finally, all statistical procedures were conducted using SPSS version 25.

## Results

### Description of overall estimations

Treatment estimations were obtained for 72 patients of the entire sample. Patients who dropped out of the study (*n* = 16) and their therapists were not evaluated. As previously reported, there were no significant differences between dropouts and completers in age (*p* = .70); sex, (*p* = .96); type of diagnoses (*p* = .35), frequency of dropouts (*p* = .22) or.Giron et al., 2023). Overall, 53 patients predicted their correct treatment type (73%), with 26 patients correctly predicting receiving OT (49%) and 27 patients correctly predicted receiving PLC (50%). On the other hand, 19 patients (26%) predicted an incorrect treatment type, with 8 patients (42%) incorrectly predicted receiving PLC while actually receiving OT, and 11 patients (58%) incorrectly predicted receiving OT while actually receiving PLC. There was no significant difference in the prevalence of accurate patient’s estimations in the OT and PLC *groups (χ*^*2*^ *= 0.27, DF = 1*, *p* = .60).

Therapist’s treatment estimations were obtained from therapists of 69 patients. Overall, 42 were found to be correct (60%), with 21 correct OT administrating estimations (50%) and 21 correct PLC administrations prediction (50%). Similarly, 27 of the therapist’s estimations (39%) were incorrect, with 13 of the patients (48%) receiving OT were predicted to receive PLC by their therapists, and 14 of the patients (52%) receiving PLC were predicted to receive OT by their therapists. Again, there was no significant difference in the prevalence of accurate therapist’s estimations across the two groups *(χ*^*2*^ *= 0.02, DF = 1*, *p* = .88).

A total of 608 estimations were provided by staff members. Overall, 65.96% of estimations of the staff were correct. Of these correct estimations, 186 correct estimations (46%) were for patients receiving OT and 215 (54%) for patients receiving PLC. Similarly, 34.04% of staff’s estimations were incorrect. Of them, 95 estimations (46%) indicated that patients received PLC while actually receiving OT, and 112 estimations (54%) indicated patients receiving OT while actually receiving PLC. There was no significant difference in the prevalence of staff accurate estimations in the OT and PLC group *(χ*^*2*^ *= 0.01, DF = 1, p = .90)*.

Associations between patient’s, therapist’s and staff’s estimations and treatment outcomes.

Overall, patient’s estimations significantly correlated with therapist’s estimations (*R* = .36, SE = 0.11, *p* < .01) and with staff’s estimations (*R* = .27, SE = 0.12, *p* < .05). However, no significant correlation was found between therapist’s and staff’s estimations.

Patient’s estimations of receiving active treatment were associated with four of the evaluated scales: OQ-45 *(B =-3.35, SE = 1.51, p < .05, R*^*2*^ *= 0.06)*, STAI-TR *(B = -1.80, SE = 0.68, p < .05 R*^*2*^ *= 0.08)*, HSCL *(B = -0.15, SE = 0.04,p < .01 R*^*2*^ *= 0.12)*, and the HAMD *(B = -1.98, SE = 0.86, p < .05, R*^*2*^ *= 0.06)*. Therapist’s estimations were associated with only two of the evaluated scales, namely OQ *(B =-4.25, SE = 1.51, p < .01, R*^*2*^ *= 0.10)* and STAI-TR *(B =-1.84, SE = 0.71, p < .05, R*^*2*^ *= 0.09)*. Staff’s estimations of active treatment were also associated with improvement in the OQ *(B =-0.58, SE = 0.26, p < .05, R*^*2*^ *= 0.07)* as well as with HSCL *(B = -0.02, SE = 0.00,p < .05, R*^*2*^ *= 0.08)*. See Table 1.

**Table 1:** Univariate linear regression analyses results for staff’s, therapist’s and patient’s estimations effects on treatment outcomes*

Staff’s estimations						Therapist’s estimations				Patient’s estimations		
	B(SE)	t	p	R^2^	B(SE)	t	p	R^2^	B(SE)	t	p	R^2^
State	-0.10 (0.22)	-0.47	0.63	0	-0.76 (1.33)	-0.57	0.56	0	-1.82 (1.27)	-1.43	0.15	0.02
anxiety												
Trait	-0.06 (0.12)	-0.54	0.58	0	**-1.84 (0.71)**	**-2.58**	**0.01**	**0.09**	**-1.80 (0.68)**	**-2.62**	**0.01**	**0.08**
anxiety												
OQ-45	**-0.58 (0.26)**	**-2.22**	**0.03**	**0.07**	**-4.25 (1.51)**	**-2.8**	**0**	**0.1**	**-3.35 (1.51)**	**-2.21**	**0.03**	**0.06**
HSCL	**-0.02 (0.00)**	**-2.41**	**0.01**	**0.08**	-0.09 (0.05)	-1.76	0.08	0.04	**-0.15 (0.04)**	**-3.16**	**0**	**0.12**
Hamilton	-0.24 (0.15)	-1.56	0.12	0.03	-0.69 (0.92)	-0.74	0.45	0	**-1.98 (0.86)**	**-2.28**	**0.02**	**0.06**

*Notes* OQ-45 - The Outcome Questionnaire-45; HSCL - Hopkins Symptom Checklist–short form; HRSD - Hamilton Rating Scale for Depression. Adjusted p-value after Bonferroni adjustment for multiple comparisons is 0.016, therefore, therapist estimations models for trait anxiety and for OQ-45 and patient estimation models for trait anxiety and HSCL remained significance after adjustments. However, staff’s estimation models for OQ-45 and HSCL and patient’s estimation models for OQ-45 and Hamilton did not

*significant results are bold

Patient’s estimations had higher predictive value then staff estimations across all measures, with standardized coefficients across scales ranging from − 0.06 to -0.26, compared with the staff’s estimations which ranged from − 0.18 to 0.07. Patient’s estimations had also a higher predictive effect as compared with therapist’s estimations in most, but not all, outcome variables. In six out of seven measures, patient’s prediction had higher predictive value as compared to therapist’s estimations, but on the OQ where therapist’s estimations coefficient *(β = -0.24, t=-1.92, p = .05)* exceeded patient’s prediction’s *(β = -0.18, t = -1.49, p = .14).* Overall, therapist prediction standardized coefficient ranged between − 0.04 to -0.24. See Table 2.

**Table 2:** Relative contribution of patient’s, therapist’s and staff’s estimations to therapy outcomes*

Staff’s estimations				Therapist’s estimation’s			Patient’s estimations		
	β	t	p	β	t	p	β	t	p
State	-0.1	-0.07	0.94	-0.02	-0.15	0.87	-0.13	-0.95	0.34
anxiety									
Trait	0.07	0.6	0.54	-0.22	-1.76	0.08	**-0.26**	**-2.02**	**0.04**
anxiety									
OQ-45	-0.14	-1.19	0.23	-0.24	-1.92	0.05	-0.18	-1.49	0.14
HSCL	-0.18	-1.5	0.13	-0.05	-0.41	0.67	**-0.26**	**-2.09**	**0.04**
HRDS	-0.12	-0.97	0.33	0.03	0.23	0.81	-0.22	-1.69	0.09

*Notes* OQ-45 - The Outcome Questionnaire-45; HSCL - Hopkins Symptom Checklist–short form; HRSD - Hamilton Rating Scale for Depression. Adjusted p-value after Bonferroni adjustment for multiple comparisons is 0.016, therefore, patient’s estimations did not remain significant after adjustments

*significant results are bold

### Associations between patient’s, therapist’s and staff’s estimations and treatment outcomes beyond study conditions

To explore whether the expectation effect sustains regardless of the presence of active treatment (i.e., whether the estimations are associated with outcomes even beyond the treatment modality), multivariate regression analyses were performed. When controlling for the actual administration of OT, the predictive effect of staff’s estimations on the OQ and HSCL reduced to become non-significant. Nonetheless, therapist’s prediction sustained its significance even after controlling for actual administration for trait anxiety (B =-1.80, t=-2.45, *p* < .05) and the OQ (B=-3.71, t=-2.41, *p* < .05). Patient’s expectation effects diminished after controlling for actual administration across two out of the four significant measures, however, sustained in trait anxiety (B = -2.02, t=-2.58, *p* < .05) and the HSCL (B = -0.13, t=-2.32, *p* < .05). See Table 3.

**Table 3:** Multiple linear regression analyses controlling for actual administration of OT*

Staff’s estinations					Therapist’s estimations				Patient’s estimations			
	B(SE)	t	P	R^2^ change	B(SE)	t	p	R^2^ change	B(SE)	t	p	R^2^ change
State anxiety	-0.17 (0.25)	-0.67	0.5	0	-0.88 (1.37)	-0.64	0.52	0	-2.45 (1.45)	-1.68	0.09	0.04
Trait anxiety	-0.04 (0.14)	-0.28	0.77	0	**-1.80 (0.73)**	**-2.45**	**0.01**	**0.08**	**-2.02 (0.78)**	**-2.58**	**0.01**	0.08
OQ-45	-0.41 (0.29)	-1.39	0.16	0.02	**-3.71 (1.53)**	**-2.41**	**0.01**	**0.07**	-2.58 (1.72)	-1.49	0.13	0.03
HSCL	-0.01 (0.01)	-1.61	0.11	0.03	-0.07 (0.05)	-1.34	0.18	0.02	**-0.13 (0.05)**	**-2.32**	**0.02**	0.06
HDRS	-0.10 (0.17)	-0.6	0.55	0	-0.28 (0.92)	-0.3	0.76	0	-1.33 (0.97)	-1.36	0.17	0.02

*Notes* OT – oxytocin; OQ-45 - The Outcome Questionnaire-45; HSCL - Hopkins Symptom Checklist–short form; HRSD - Hamilton Rating Scale for Depression. Adjusted p-value after Bonferroni adjustment for multiple comparisons is 0.016, therefore, therapist and patient’s estimation models for trait anxiety remained significance after adjustments, whereas therapist’s estimation model for OQ-45 and patient’s estimation model for HSCL did not

*significant results are bold

## Discussion

The present study examined the influence of staff’s, therapist’s, and ‘ estimations of treatment type (active/placebo) on the reinforcing effect of oxytocin. To our knowledge, this is the first attempt to assess the impact of patient’s, therapist’s, and staff’s expectations, reflected in estimations of treatment type, on treatment outcome in an inpatient psychiatric facility.

Our results suggest that staff’s estimations have an effect on overall distress and symptom relief. However, this effect disappears when the actual administration of OT is taken into account. It is possible that the therapeutic atmosphere may enhance or attenuate the effect of active treatment but, beyond that, may not produce an inherent placebo effect. Additionally, the nature of the therapeutic alliance and the relatively mild emotional interaction between the staff and the patient might have generated a mild placebo effect that did not exceed the effect of the substance administered. Previous studies have shown that staff attitudes toward patients vary widely, particularly toward difficult patients who present challenges to staff in terms of suicidality, self-mutilation, mood instability, impulsivity, and sense of entitlement(Bodner et al. 2011, 2015a, b). Of course, negative staff attitudes can distort or diminish positive expectations regarding a patient. Because this study is the first to examine placebo effects in the inpatient setting, further studies can disentangle positive and negative staff expectations to explore how each produces an expectancy effect.

Therapist’s estimations maintained their significant effect beyond OT treatment on inpatient’s anxiety and general distress, suggesting that the personal therapist may enhance a placebo effect beyond active treatment. Previous studies have clearly demonstrated the impact of therapist’s expectations on therapeutic outcomes, but mainly in the outpatient setting (Glick and Margolis 1962; Jacob 1971; Rutherford et al. 2010b; Howe et al. 2017). The results of the current study support these findings while also extending knowledge of therapist’s expectations in the inpatient setting. It appears that in the context of the intensive dyadic therapeutic relationship, the expectations of the personal therapist produce a stronger placebo effect than staff (Coppock et al. 2010). Studies suggest that therapist’s hopes for treatment success are related to treatment outcomes. The results of the current study suggest that this prediction extends even beyond the effects of the treatment itself, highlighting the importance of therapist’s-related factors on treatment outcome. The impact of therapist’s expectations on anxiety levels is supported by the fact that trait anxiety responds to various stimuli such as exercise, nature walks, and sleep deprivation and can be reduced in children in the context of a good therapeutic alliance (Marker et al. 2013; Ensari et al. 2015; Pires et al. 2016; Kotera et al. 2021). Other studies demonstrate that warm and empathetic communication combined with positive expectations can also reduce the level of anxiety in adults(Verheul et al. 2010).

Patient’s estimations also had an impact on treatment outcome, and this effect persisted beyond the actual administration of OT, in terms of anxiety levels and symptom relief. These results suggest that the expectations of severely distressed patients in the psychiatric ward are important in eliciting a placebo response even beyond the effects of the treatment itself. Interestingly, therapist’s expectations enhance a more general placebo effect (reduction of general distress and anxiety), whereas patient’s expectations elicit a more specific placebo effects that focuses on particular symptoms that patients are struggling with. This is consistent with the study by Benedetti et al. which showed that the expectation of pain relief in a particular organ produced an analgesic placebo effect specifically in that organ(Benedetti et al. 1999). It is possible that this can be generalized to the placebo response in psychiatric treatments. This underscores the efficacy of patient’s expectations and the importance of inducing a symptom-specific expectation in the patient’s consciousness.

This study has several limitations. Self-report questionnaires were mainly used to assess the results. In addition, patient’s diagnosis, pharmacological treatment, and support system may have influenced their expectations for relief and the extent of their placebo response. Although awareness of receiving active treatment is clearly an essential element for patient’s expectations and their placebo response, another limitation of the study is that expectations were assessed indirectly via estimations regarding active/placebo treatment, and pre-trial expectations were not collected. Lack of data regarding the estimations of patients who dropped out is another limitation of the study.

Sample size should be increased in future studies to further assess the reported effects while controlling for multiple comparisons. Nonetheless, knowing, or at least believing, that one is receiving an active treatment generates high levels of expectancy, and has a positive impact on placebo effects and therapeutic outcome(Sotsky et al. 1991; Mondloch et al. 2001; de la Fuente-Fernández et al. 2001; Petrovic et al. 2002; Kaptchuk et al. 2008). Finally, oxytocin itself may play a central role in the neurobiological mechanism of the placebo response, which may also influence our results(Enck and Klosterhalfen 2009).

The present study attempts to distinguish between three levels of expectancy related to the placebo response in the psychiatric ward: the patient, his or her primary caregiver, and the staff. The results of the study highlight the great importance of the therapist’s expectations as a factor that can produce therapeutic effects beyond the treatment modality. Furthermore, it highlights the importance of examining both patient’s and therapist’s expectations and their influence on placebo effects in future studies. This is particularly important for future studies exploring neurobiological facilitators of therapeutic effects that are currently prominent in psychiatric research, such as ketamine, psilocybin, and MDMA(Carhart-Harris et al. 2017; Smith et al. 2022; Smith-Apeldoorn et al. 2022). We address the impact of treatment expectations in the context of the psychiatric ward and provide preliminary insight into the potential role of staff expectations in determining treatment outcomes. Further research is needed to assess the clinical relevance of different levels of expectations and to understand the biological and psychological mechanisms underlying the placebo response.
